# Effects of Cu Substituting Mo in Sr_2_Fe_1.5_Mo_0.5_O_6−δ_ Symmetrical Electrodes for CO_2_ Electrolysis in Solid Oxide Electrolysis Cells

**DOI:** 10.3390/nano15080585

**Published:** 2025-04-11

**Authors:** Wanting Tan, Pengzhan Hu, Tianxiang Feng, Siliang Zhao, Shuai Wang, Hui Song, Zhaoyu Qi, Wenjie Li

**Affiliations:** 1 School of Ecology and Environment, Zhengzhou University, Zhengzhou 450001, China; 2 College of Chemistry, Zhengzhou University, Zhengzhou 450001, China; 3 Key Laboratory of Environmental Protection in Water Transport Engineering Ministry of Transport, Tianjin Research Institute for Water Transport Engineering, Tianjin 300456, China; 4 Henan Key Laboratory of Environmental Chemistry and Low Carbon Technology, Zhengzhou 450001, China

**Keywords:** CO_2_, Sr_2_Fe_1.5_Mo_0.5_O_6−δ_, perovskite, solid oxide electrolysis cells, Cu substitution

## Abstract

Solid oxide electrolysis cells (SOECs) are considered one of the most promising technologies for carbon neutralization, as they can efficiently convert CO_2_ into CO fuel. Sr_2_Fe_1.5_Mo_0.5_O_6−δ_ (SFM) double perovskite is a potential cathode material, but its catalytic activity for CO_2_ reduction needs further improvement. In this study, Cu ions were introduced to partially replace Mo ions in SFM to adjust the electrochemical performance of the cathode, and the role of the Cu atom was revealed. The results show Cu substitution induced lattice expansion and restrained impurity in the electrode. The particle size of the Sr_2_Fe_1.5_Mo_0.4_Cu_0.1_O_6−δ_ (SFMC0.1) electrode was about 500 nm, and the crystallite size obtained from the Williamson–Hall plot was 75 nm. Moreover, Cu doping increased the concentration of oxygen vacancies, creating abundant electrochemical active sites, and led to a reduction in the oxidation states of Fe and Mo ions. Compared with other electrodes, the SFMC0.1 electrode exhibited the highest current density and the lowest polarization resistance. The current density of SFMC0.1 reached 202.20 mA cm^−2^ at 800 °C and 1.8 V, which was 12.8% and 102.8% higher than the SFM electrodes with and without an isolation layer, respectively. Electrochemical impedance spectroscopy (EIS) analysis demonstrated that Cu doping not only promoted CO_2_ adsorption, dissociation and diffusion processes, but improved the charge transfer and oxygen ion migration. Theory calculations confirm that Cu doping lowered the surface and lattice oxygen vacancy formation energy of the material, thereby providing more CO_2_ active sites and facilitating oxygen ion transfer.

## 1. Introduction

The extensive use of fossil fuels has caused a dramatic increase in CO_2_ emissions. To achieve carbon neutrality, it is crucial to develop effective technologies to reduce and utilize CO_2_. Various CO_2_ reduction and conversion technologies have been developed, including photocatalysis [1,2], thermocatalysis [3,4], and electrocatalysis [5].

Photocatalysis is a technology that uses light energy to drive the reduction reaction of CO_2_. Its core principle is that photosensitizers or semiconductor materials absorb photons, generating electron–hole pairs, which then catalyze the conversion of CO_2_ into hydrocarbons or other high-value-added chemicals [6]. Current research focuses on strategies to enhance photocatalytic efficiency, including heterojunction construction, defect engineering, single-atom catalysts, and cocatalyst loading. Photocatalysis offers advantages such as direct solar energy utilization, mild reaction conditions, and environmental friendliness. However, its limitations include low quantum efficiency, poor product selectivity, and insufficient catalyst stability [7,8]. Thermocatalysis primarily employs high temperatures and catalysts (Cu/ZnO, Fe-based, Co-based) to hydrogenate CO_2_ into high-value chemicals such as CH4, methanol, and light olefins. The fundamental principle involves the adsorption and activation of CO_2_ and H_2_ on catalyst active sites, followed by hydrogenation reactions that cleave C=O bonds and form C-H bonds [9]. Contemporary research efforts are primarily directed toward sophisticated catalyst engineering strategies, aiming to enhance both catalytic activity and product distribution control. Thermocatalysis offers rapid reaction rates and high product yields, making it suitable for large-scale industrial applications. However, it requires harsh conditions (high temperature and pressure) and suffers from catalyst deactivation (coking or sintering) [10,11]. Electrocatalysis employs electrical energy to drive catalysts for converting CO_2_ into value-added chemicals such as CO, formate, ethylene, and ethanol. The core mechanism involves electrochemical reactions in which CO_2_ molecules accept electrons at the cathode surface, undergoing a series of intermediate steps to form target products [12]. Electrocatalytic CO_2_ reduction offers significant advantages, including tunable reaction pathways via applied voltage and temperature control, compatibility with renewable energy sources (wind, solar, hydro, and geothermal power), and the potential to establish a carbon-neutral energy cycle [13]. Moreover, it provides an effective means of storing intermittent renewable energy in chemical form.

Solid oxide electrolysis cells (SOECs) are an effective CO_2_ reduction technology and energy conversion device [14]. With external electrical power, SOECs can convert CO_2_ into CO, which can be used as an industrial feedstock. More importantly, they can directly use renewable energy as power, offering the potential to close the CO_2_ loop and achieve carbon neutrality [13]. The cell structure consists of three components: the porous cathode, anode, and sandwiched solid electrolyte. During operation, CO_2_ gas is adsorbed at the cathode and reduced by receiving electrons to generate CO and O^2−^. The O^2−^ ions migrate through the solid electrolyte to the anode, where they release electrons to generate O_2_. In these reactions, the reduction of CO_2_ at the cathode is the key rate-limiting step. Thus, developing high-performance cathode materials is indispensable for the efficient operation of SOECs [15].

Nickel-yttria-stabilized zirconia (Ni-YSZ) is a widely studied and used traditional cathode material for SOECs due to its excellent electrocatalytic activity and relatively low cost [16]. However, Ni-YSZ still faces numerous challenges when electrolyzing pure CO_2_ at high temperatures. For instance, its redox stability is poor, as it can be oxidized to NiO, which lowers the conductivity of the electrode [17]. Additionally, carbon deposition in pure CO_2_ atmospheres can block active reaction sites [18], and Ni particles within the composite tend to agglomerate and coarsen [19]. As a result, the development of nickel-free, high-performance cathode materials has become a significant research focus. Perovskite materials, due to their high ionic conductivity, stable redox properties, and good resistance to carbon deposition, have received extensive attention in recent years and are considered one of the most promising candidates for SOEC cathodes [13]. For example, Sr_2_Fe_1.5_Mo_0.5_O_6−δ_ (SFM) [15], La_0.75_Sr_0.25_Cr_0.5_Mn_0.5_O_3−δ_ [20], La_0.67_Sr_0.33_Fe_0.67_Ti_0.33_O_3−δ_ [21], and Pr_0.4_Sr_0.6_Fe_0.9_Mo_0.1_O_3_ [22] have been reported as cathode materials for CO_2_ electrolysis in SOECs. Among these perovskite materials, SFM has attracted significant interest because of its high conductivity in both cathode and anode atmospheres, making it a promising candidate for symmetric electrodes in SOECs and offering a potential for reducing fabrication costs [19]. However, the catalytic activity of traditional SFM materials for CO_2_ reduction still requires further improvement.

In recent years, various strategies have been developed to enhance the catalytic activity of SFM for CO_2_ electrolysis, including impregnation [23,24], in situ dissolution [24,25], and doping. Doping other ions into the A-site, B-site, or even the O-site of the material can regulate the oxygen vacancy content and is an effective strategy to enhance cathode performance. For example, Yang et al. (2023) found that doping Bi into SFM improved CO_2_ adsorption on the electrode surface and enhanced the electron conductivity within the electrode, thereby improving the CO_2_ electrolysis performance [15]. Sun et al. (2022) discovered that doping La into SFM promoted the surface exchange and bulk-phase diffusion of oxygen species [26]. Li et al. (2019) doped F ions into SFM, finding that F ions nearly doubled the CO_2_ adsorption capacity and increased the concentration of bulk-phase oxygen vacancies by 35–37%, thus enhancing the surface CO_2_ reduction reaction rate and ion diffusion in the bulk phase [27]. Liu et al. (2025) doped Sc into the B-site of SFM, which increased the concentration of oxygen vacancies and improved CO_2_ adsorption ability, thereby increasing the current density and reducing the polarization resistance of SOECs [28]. Xi et al. (2021) reported Mg-doped SFM materials, finding that an appropriate amount of Mg doping not only improved redox stability but also lowered the oxygen vacancy formation energy, thereby promoting CO_2_ electrolysis [17].

Cu is a popular transition metal in the field of catalysis, and as a dopant, it can enhance various properties of perovskite materials. For instance, Lim et al. (2024) doped Cu ions into Ba_0.5_Sr_0.5_FeO_3−δ_ for use as a cathode material in solid oxide fuel cells and found that Cu doping increased the oxygen vacancy concentration and improved surface exchange and bulk-phase transport of oxygen ions [29]. In our previous work, Cu-doped La_0.8_Sr_0.2_MnO_3_ was constructed for the electrochemical reduction of NO_x_, and it was found that Cu doping introduced more oxygen vacancies and decreased the particle size, thus increasing the NO_x_ removal efficiency [30]. Tailor et al. (2023) studied the effect of Cu doping in lead-free halide perovskites on photocatalytic CO_2_ reduction performance, finding that Cu doping slowed the thermal carrier relaxation and extended the carrier decay lifetime, thus enhancing the charge migration efficiency and improving CO_2_ reduction performance [31]. Berger et al. (2024) investigated Cu-doped perovskite-type oxides (Nd_0.6_Ca_0.4_Fe_1−x_Cu_x_O_3_ and Pr_0.6_Ca_0.4_Fe_1−x_Cu_x_O_3_) as catalysts for Methanol Steam Reforming. The results demonstrated that Cu-doped perovskite-type oxides with tailored A-site and B-site compositions promote the formation of Cu nanoparticles, highlighting the importance of composition optimization for designing efficient catalytic systems [32]. Derakhshi et al. (2024) investigated the effect of Cu substitution for Fe in LaFe_1−x_Cu_x_O_3_ perovskite nanostructures for detecting volatile organic compounds (VOCs). The results show significant improvements in gas-sensing properties, with enhanced responses to ethanol, decreased nanoparticle sizes, and increased electrical conductivity [33]. Zhang et al. (2022) developed a Cu-doped perovskite oxide, CaFe_0.9_Cu_0.1_O_3_, as a cathode electrocatalyst for microbial fuel cells (MFCs). The results show that this catalyst outperformed Pt/C in terms of lower overpotential, better stability, and superior power density, making it an efficient and cost-effective alternative for MFC applications [34]. Xu et al. (2019) replaced part of the Fe ions in SFM with Cu ions and found that Cu substitution enhanced the CO_2_ adsorption capacity of material at high temperatures and reduced the electrode interface polarization resistance [35]. However, to our best knowledge, there is no study on the effect of Cu ion substitution for Mo in SFM, nor has the micro-mechanism of Cu doping on the electrochemical CO_2_ reduction performance of the electrode been thoroughly investigated. This study aims to develop a novel high-performance SOEC cathode material by substituting Mo sites in SFM perovskite with Cu. The work will elucidate the regulation mechanism of Cu doping on electrocatalytic activity, providing a new strategy for designing efficient and stable cathodes for solid oxide electrolysis cells.

In this work, for the first time, we employed Cu ions to replace Mo in SFM as a symmetric electrode for CO_2_ electrolysis and examined how Cu doping concentration influences the electrochemical performance of SFM electrodes. In addition, we investigated the regulatory mechanism of Cu ions on the electrode performance. The crystal composition, microstructure, and surface elemental valence states of the materials and electrodes were studied by systematic characterization. Electrochemical tests were performed to evaluate the current density and electrochemical impedance spectra (EIS) at different voltages of the electrodes, and CO_2_ reduction processes were identified through distributed relaxation time (DRT) analysis. Combined with density functional theory (DFT) calculations, the impact of Cu ion doping on the material properties was further analyzed, thus revealing the fundamental role of Cu ions in performance improvement at the atomic level. This study systematically elucidates the influence mechanism of Cu doping on electrode material performance, providing important theoretical foundations and methodological references for the design of high-performance SOEC electrodes, thereby further enriching the research framework in this field.

## 2. Experiment

### 2.1. Powders Preparation

**Figure 1:**
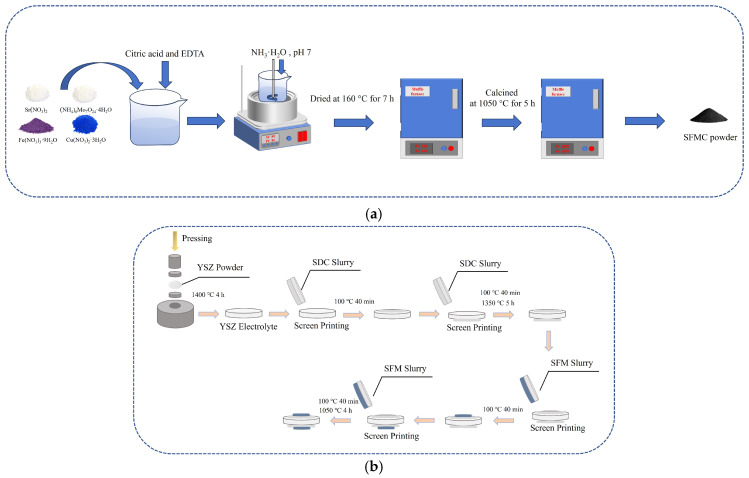
Schematic diagram of (**a**) SFMC powder synthesis and (**b**) cell fabrication..

The Sr_2_Fe_1.5_Mo_0.5−x_Cu_x_O_6−δ_ (SFMC) powders (with x = 0, 0.1, and 0.3, denoted as SFM, SFMC0.1, and SFMC0.3, respectively) were synthesized using the citric acid-EDTA method [36]. Briefly, stoichiometric amounts of Sr(NO_3_)_2_, Fe(NO_3_)_3_·9H_2_O, (NH_4_)_7_Mo_7_O_24_·4H_2_O, and Cu(NO_3_)_2_·3H_2_O were dissolved in a small amount of deionized water, and then citric acid and EDTA were added. The molar ratio of the total metal ions, EDTA and citric acid was controlled at 1: 1: 1.5. The solution above was stirred in a water bath at 80 °C, while ammonia solution was slowly added to adjust the pH to around 7. As the solution evaporated, a brownish wet gel formed. The wet gel was then transferred to a muffle furnace and dried at 160 °C for 7 h, resulting in a foamy black dried gel. This dried gel was calcined in a muffle furnace at 1050 °C for 5 h to obtain a fluffy, flocculent product, which was then ground to obtain the SFMC powders. The ionic conductor Ce_0.8_Sm_0.2_O_1.9_ (SDC) powder was also prepared using a similar citric acid-EDTA method, but the nitrates used were Ce(NO_3_)_3_·6H_2_O and Sm(NO_3_)_3_·6H_2_O, and the calcination temperature was 900 °C. Figure 1a shows the schematic diagram of SFMC powder synthesis.

### 2.2. Cell Fabrication

Preparation of YSZ electrolyte: 200 mg of YSZ powder was placed into a 13 mm diameter die and pressed at 200 MPa to form a pellet-shaped green body for the electrolyte. The green body was sintered in a high-temperature furnace at 1400 °C for 4 h to obtain a dense YSZ electrolyte substrate. The thickness of the sintered YSZ electrolyte substrate was approximately 400 μm.

Preparation of SDC isolation layer: To prevent potential chemical reactions between the electrode materials and YSZ, an SDC isolation layer was applied to both sides of the YSZ electrolyte. Briefly, SDC powder was mixed with turpentine in a 1: 1.5 mass ratio, ground to a viscous slurry, and then the slurry was screen-printed onto both sides of the YSZ electrolyte. The coated electrolyte was then sintered at 1350 °C for 5 h.

Preparation of SFMC/SDC electrode layer: SFMC powder was mixed with SDC powder and turpentine in a 65:35:150 mass ratio and thoroughly ground to obtain a composite electrode slurry. The slurry was then screen-printed onto both sides of the YSZ electrolyte, which had the SDC isolation layer. The electrodes were sintered at 1050 °C for 4 h. After these steps, a single cell was assembled with YSZ as the electrolyte, SDC as the isolation layer, and SFMC/SDC as the composite symmetric electrode. To verify the role of the SDC isolation layer, another cell using SFM without the SDC isolation layer was prepared, denoted as SFM’. Figure 1b shows the schematic diagram of the cell fabrication.

### 2.3. Physical and Chemical Characterization

The crystal structure of the synthesized powders and cells was characterized by X-ray diffraction (XRD, D8 Advance, Bruker, Germany). The 2θ range was from 5° to 90°, and the scanning speed was 10° min^−1^. The microstructure of the electrodes and cells was observed using a field emission scanning electron microscope (SEM, JEOL-S4800, Tokyo, Japan and ZEISS-300, Oberkochen, Germany) and transmission electron microscope (TEM, JEOL JEM-F200, Tokyo, Japan). Elemental distribution analysis was performed using energy-dispersive spectroscopy (EDS) attached to the SEM. X-ray photoelectron spectroscopy (XPS, Thermo Scientific K-Alpha, Waltham, MA, USA) was used to analyze the chemical states of elements on the surface of the electrode powders. To eliminate charge effects and instrument drift, the C 1s peak was calibrated to 284.8 eV.

### 2.4. Electrochemical Performance Testing

Silver wires were connected to the electrodes using conductive silver paste, and the cell was sealed at one end of an alumina tube with high-temperature adhesive. The alumina tube was then placed in a custom-made tube furnace for testing, where the cathode was exposed to a pure CO_2_ atmosphere while the anode was exposed to air. The CO_2_ gas flow rate was set to 50 mL min^−1^ using a mass flow meter. EIS plots of the cell were measured in the temperature range of 650–800 °C using an electrochemical workstation (CHI 660E, Austin, TX, USA). The frequency range for EIS measurement was 0.1–10^6^ Hz, with a voltage amplitude of 10 mV. The obtained EIS spectra were analyzed using DRT to identify and understand different electrochemical processes. The current–voltage (I-V) characteristics of the cell were measured using linear sweep voltammetry (LSV). The short-term stability of the cell was tested using a chronoamperometry method. The CO production rate at the outlet was measured using a gas analyzer (TESTO 330, Lenzkirch, Germany), and the Faradaic efficiency (FE) of the cell was calculated using the following formula:

FE = R′_CO_/R_CO_ (1)

where R′_CO_ is the experimentally measured CO production rate, and R_CO_ is the theoretical CO production rate, which can be calculated using the formula [37]:

R_CO_ = nV_m_ = (ItV_m_)/(Fz) (2)

where n is the number of moles of CO produced, I is the current density, t is the electrolysis time, F is Faraday’s constant (96,485 C mol^−1^), z is the number of electrons transferred in CO_2_ reduction, and V_m_ is the molar volume of the gas.

### 2.5. Computational Details

The DFT calculation was conducted based on CASTEP code. The generalized gradient approximation (GGA) with the Perdew–Burke–Ernzerhof (PBE) exchange correlation density functional was used, and all the calculations were spin-polarized. The simplified cell unit of Sr_2_FeMoO_6_ with 40 atoms and a cubic structure was used for basic bulk calculation. Based on the optimized structure of bulk, a slab containing five-layer atoms (52 atoms) with a 15 Å vacuum layer was built for surface calculation. The top three layers were fully relaxed and the remaining two layers were constrained for geometry optimization. Since it is widely accepted that B-site atoms dominate active molecules, only the B-O terminated structure was investigated. To model the Cu-doped SFM, one surface Mo atom was substituted by a Cu atom. The cutoff energy of 571.4 eV and SCF tolerance of 10^−6^ eV atom^−1^ was used for calculation. To better describe the structure properties, the Hubbard correlation (U) of 4.0 eV was applied in an Fe atom. The k-point grids of 2 × 2 × 2 and 2 × 2 × 1 were used for bulk and slab calculations, respectively. The geometry optimization was finished as the energy, force and displacement were simultaneously converged to a 10^−5^ eV atom^−1^, 0.03 eV Å^−1^ and 0.001 Å, respectively. The oxygen vacancy formation energy (E_f_) was calculated according to the following formula [38]:

E_f_ = E_def_ − E_per_ + 1/2E_oxygen_ (3)

where E_def_ and E_per_ are the total energies of the defect structure, with one oxygen vacancy, and the perfect structure, respectively. E_oxygen_ is the total energy of an oxygen molecule.

## 3. Results and Discussion

### 3.1. Crystal Structure and Morphology

**Figure 2:**
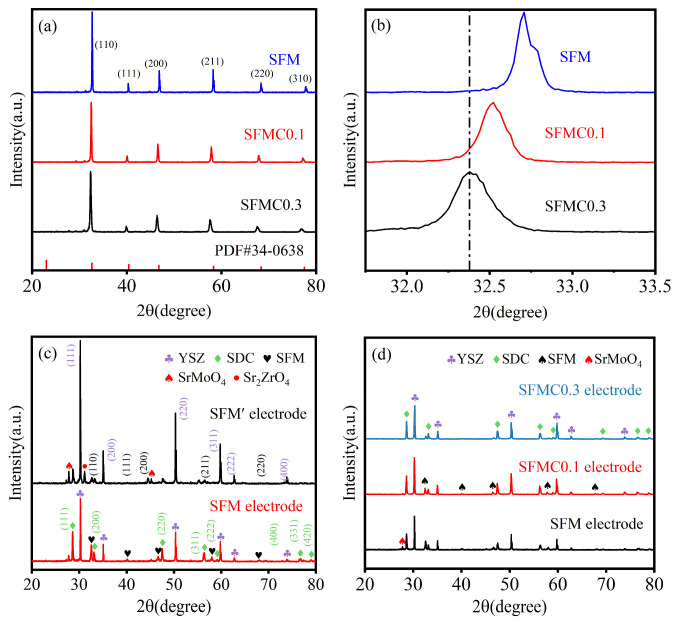
(**a**) XRD pattern of SFM, SFMC0.1, and SFMC0.3. (**b**) Enlarged XRD pattern in the 2θ range of 30–35°. (**c**) XRD pattern of electrodes with and without the SDC isolation layer. (**d**) XRD spectra of SFM, SFMC0.1, and SFMC0.3 electrodes.

Figure 2a shows the XRD spectra of the SFM powders with different Cu doping levels. As shown in the picture, the main peaks of all the samples match the diffraction peaks of the standard PDF card (PDF#34-0638, cubic structure, Pm-3m space group), indicating the successful synthesis of the SFM double perovskite structure. As displayed in the enlarged XRD pattern in Figure 2b, with increasing Cu content, the diffraction peaks exhibit weakened intensity and a gradual shift toward lower angles, indicating that Cu doping reduced crystallinity and induced lattice expansion. This lattice distortion weakened the Mo-O bonds and lowered the formation energy of oxygen vacancies. The direct cause of this phenomenon is that the ionic radius of Cu^2+^ (0.073 nm) is larger compared to Mo^6+^ (0.059 nm) and Mo^5+^ (0.061 nm) [39,40]. In addition, Cu^2+^ doping may also lead to a reduction in the oxidation states of Fe and Mo ions, which could further increase the crystal volume, as explained in the following XPS analysis. The Rietveld refinement results are shown in Figure S1 and Table S1 in the Supporting Information. Samples exhibit a cubic perovskite structure with the space group of Pm-3m. As shown in Figure S2, the Williamson–Hall (W-H) graph was given to determine the crystallite size. According to the results (Table S2), the crystallite size for SFM and SFMC0.1 is 98.3 and 75.4 nm, respectively, indicating that moderate Cu doping decreased the crystallite size. However, the crystallite size of SFMC0.1 obtained from the Scherrer equation was 32.5 nm, which was smaller than the size obtained from the W-H graph. This difference indicates the presence of a substantial micro-strain in the SFM perovskite, as the Scherrer equation solely attributes peak broadening to crystallite size, while the W-H method accounts for both size and strain effects, revealing that strain-induced broadening artificially reduces the Scherrer-derived size [41].

To investigate the chemical compatibility between the electrode material and the electrolyte, Figure 2c shows the XRD spectra of the SFM electrodes with and without the SDC isolation layer. The electrode without the SDC isolation layer (SFM’), in addition to the diffraction peaks of SFM, SDC, and YSZ, also shows impurity phases, such as SrMoO_4_ and Sr_2_ZrO_4_. The SrMoO_4_ impurity often appears during the synthesis of Mo-based perovskite oxides. According to the literature, SrMoO_4_ impurity likely arose from the melting of intermediate MoO_3_ oxides [42,43]. The Sr_2_ZrO_4_ phase is a product of the reaction between SFM and YSZ, which can significantly affect the electrode’s performance [44]. After adding the SDC isolation layer, the intensity of the Sr_2_ZrO_4_ diffraction peaks significantly decreases, confirming the necessity of adding the SDC isolation layer. Therefore, the electrodes with a SDC isolation layer are used for subsequent studies. Figure 2d shows the XRD pattern of the electrodes with different Cu doping contents. It is observed that the SrMoO_4_ and Sr_2_ZrO_4_ impurity phases disappear after Cu doping, and no new phases form, probably indicating that the chemical compatibility between the electrode and the electrolyte is improved.

**Figure 3:**
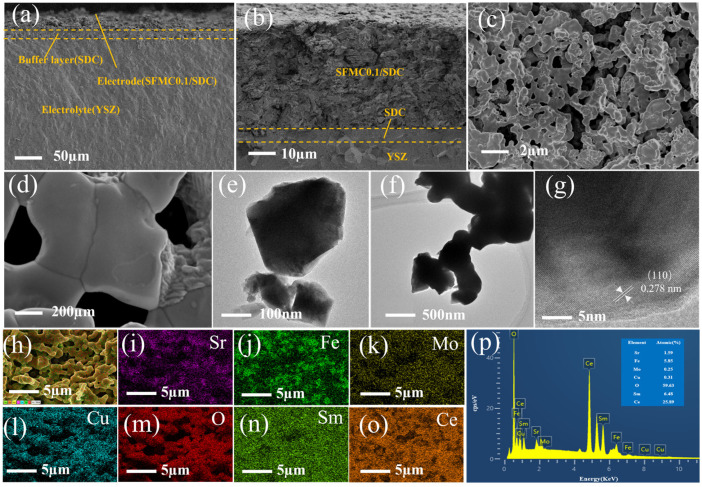
(**a**,**b**) Cross-sectional SEM images, (**c**,**d**) surface SEM images, and (**e**–**g**) TEM images of the SFMC0.1 electrode. (**h**–**o**) Elemental mapping of the electrode surface, (**p**) EDS of the electrode surface.

Figure 3a,b show the cross-sectional SEM images of the cell. The images reveal a dense electrolyte and a loose, porous electrode. The SDC isolation layer is located between the electrolyte and the electrode, preventing the side reactions between the electrode material and the electrolyte. The electrode, isolation layer, and electrolyte are tightly connected, facilitating the transport of oxygen ions from the composite electrode to the electrode. Figure 3c,d show the surface SEM images of the SFMC0.1 electrode. As shown in the images, the electrode surface has a porous structure, which is beneficial for the diffusion and adsorption of gas molecules. The particles are tightly connected, facilitating the migration of electrons and O^2−^ ions. The particle size on the surface of the SFMC0.1 electrode was approximately 500 nm. Figure 3e–g show the TEM image of SFMC0.1. The distance between the two parallel planes is 0.278 nm, corresponding to the (110) plane of SFMC0.1 perovskite. Figure 3h–o show the elemental mapping of the SFMC0.1 electrode surface. The distributions of Cu elements match those of Sr, Fe, and Mo, confirming the successful doping of Cu into the SFM. The distribution of Ce and Sm elements differs from that of the SFMC components, proving that the SDC and SFMC components are interspersed, which expands the three-phase reaction boundary. The EDS results shown in Figure 3p are in good agreement with the stoichiometry of Sr_2_Fe_1.5_Mo_0.4_Cu_0.1_O_6−δ_-Ce_0.8_Sm_0.2_O_1.9_, further confirming the successful preparation of the electrode material.

### 3.2. XPS Analysis

**Figure 4:**
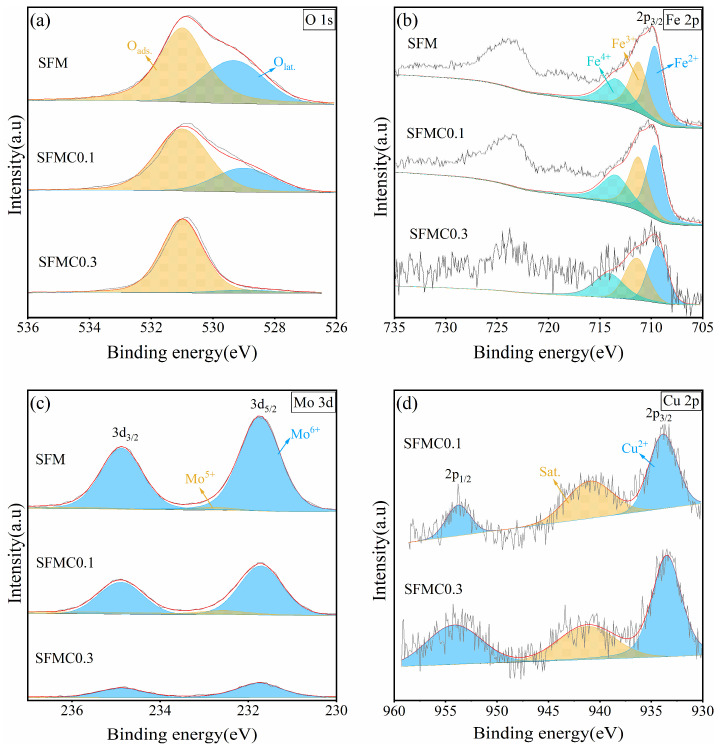
XPS spectra of SFM, SFMC0.1, and SFMC0.3 powders: (**a**) O 1s, (**b**) Fe 2p, (**c**) Mo 3d, (**d**) Cu 2p.

As shown in Figure 4a, the XPS spectra of O 1s can be divided into two peaks. The peak at the lower binding energy (around 529.8 eV) corresponds to lattice oxygen (O_lat_), while the peak at the higher binding energy (around 531.0 eV) corresponds to adsorbed oxygen species [15]. It is widely believed that the content of adsorbed oxygen reflects the surface oxygen vacancy concentration [45]. According to the fitting results (Table 1), the adsorbed oxygen ratio in SFMC0.1 is 72.1%, which is significantly higher than the 63.1% in SFM. This phenomenon indicates that Cu doping increases the surface oxygen vacancies in the material. According to related literature, CO_2_ reduction reaction kinetics are closely related to the surface oxygen vacancy concentration of the electrode, as oxygen vacancies can serve as hosts for CO_2_ molecules, which is crucial for the chemical adsorption of CO_2_ at high temperatures [15].

Figure 4b shows the XPS spectra of Fe 2p. The Fe 2p spectrum exhibits two asymmetric peaks, with the lower binding energy region corresponding to the 2p_3/2_ orbital. The binding energies around 709.7 eV, 711.2 eV, and 713.5 eV correspond to Fe^2+^, Fe^3+^, and Fe^4+^ in the 2p_3/2_ orbital, respectively [38]. According to the fitting results (Table 2), Cu doping causes Fe ions to shift toward lower oxidation states, increasing the Fe^2+^ content while decreasing the Fe^3+^ and Fe^4+^ content.

As shown in Figure 4c, the signal intensity of Mo decreases as the Cu substitution amount increases. The Mo 3d spectrum shows two peaks, with binding energies around 232.8 eV and 231.7 eV corresponding to Mo^6+^ and Mo^5+^, respectively [38]. According to the fitting results (Table 3), Mo ions primarily exist in the Mo^6+^ form, and after Cu doping, the content of Mo^5+^ increases, indicating a slight reduction in the overall oxidation state of Mo ions. The presence of mixed valence Mo^5+^/Mo^6+^ can enhance the ionic conductivity and catalytic activity required for the CO_2_ electrolysis process. According to the principle of charge neutrality, higher concentrations of low-valence Fe^2+^ and Mo^5+^ ions result in increased oxygen vacancy formation. Therefore, the XPS results for Fe and Mo are generally consistent with the O 1s results.

XPS analysis demonstrates that Cu doping increases Fe^2+^/Fe^3+^ and Mo^5+^/Mo^6+^ redox couples, promoting polaron hopping and enhancing electronic conductivity. The increase in oxygen vacancy concentration (verified by the increase in oxygen defects proportion) provides a channel for O^2−^ migration and reduces the migration activation energy of oxygen ions. Cationic defects introduced by Cu doping work synergistically with oxygen vacancy to form local charge delocalization states (such as Cu-3d/O-2p hybridization) and improve intrinsic conductivity, which can be verified by the DOS analysis [46].

Figure 4d shows the XPS spectrum of Cu 2p in the electrode material. The Cu 2p_3/2_ spectrum displays two peaks: the peak at 933.48 eV corresponds to Cu^2+^, and the peak at 941.26 eV corresponds to the satellite peak. It can be identified that Cu in SFMC is mainly in the form of Cu^2+^ [39].

### 3.3. Electrochemical Performance

**Figure 5:**
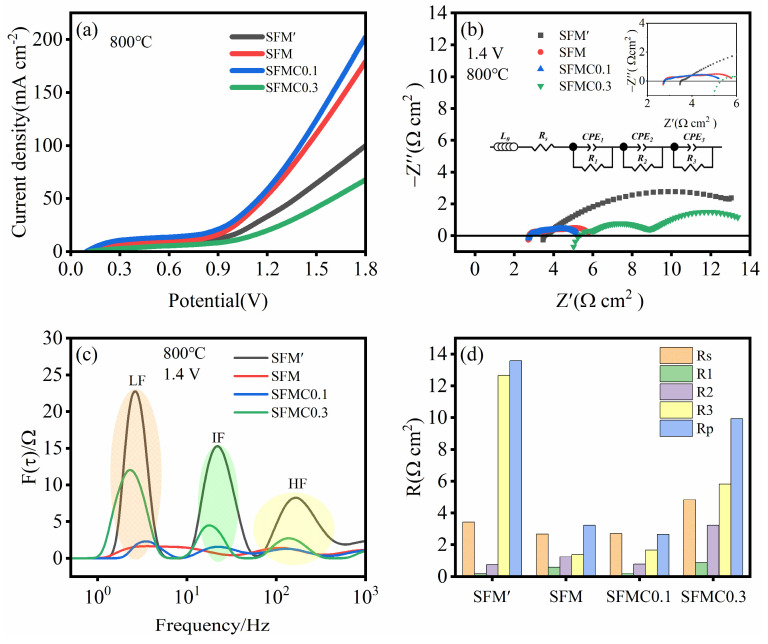
Electrochemical performance of cells based on different electrode materials. (**a**) I-V curves, (**b**) EIS curves at 1.4 V, (**c**) corresponding DRT spectra, and (**d**) fitted resistance values.

Figure 5a presents the I-V curves of cells based on different electrode materials. It can be observed that the introduction of an isolation layer significantly increases the current density, and appropriate Cu doping further enhances the current density. At 1.8 V, the cell based on SFMC0.1 shows the highest current density of 202.20 mA cm^−2^, followed by SFM (179.26 mA cm^−2^), SFM’ (99.68 mA cm^−2^) and SFMC0.3 (67.73 mA cm^−2^). Figure 5b shows the EIS of different electrodes. The EIS spectra for each electrode consist of several semi-circles. In general, the high-frequency portion of the EIS spectrum and its intersection with the *x*-axis represent the ohmic resistance, while the low-frequency portion and its intersection with the *x*-axis represent the total resistance. The difference between the total resistance and the ohmic resistance represents the polarization resistance of the electrode [47]. As shown in Figure 5b, the SFMC0.1 cell exhibits the smallest total and polarization resistances, while excessive Cu doping results in a significant increase in resistance for the SFMC0.3 cell.

To further investigate the electrochemical reaction process, the electrochemical impedance spectra were analyzed using an equivalent circuit model: L_0_R_s_(R_1_-CPE_1_)(R_2_-CPE_2_)(R_3_-CPE_3_) via the DRT technique [26]. In the model, L represents inductance, and Rs represents the total ohmic resistance related to the interface, including electrolyte, electrode and contact resistance. R_1_, R_2_, and R_3_ represent the polarization resistances in the high-, medium-, and low-frequency regions, while CPE_1_, CPE_2_, and CPE_3_ are constant phase elements corresponding to the respective frequency regions. As shown in Figure 5c, the DRT spectrum can be divided into high-, medium- and low-frequency regions. The high-frequency region (HF > 100 Hz) is likely associated with oxygen ion transfer steps between the YSZ electrolyte and electrode, while the intermediate-frequency region (IF) may relate to charge transfer, and the low-frequency region (LF < 10 Hz) is attributed to the surface kinetics of the fuel electrode, including adsorption, activation, diffusion and dissociation processes of the active species [25]. The DRT results demonstrate that the peak areas corresponding to the HF and LF regions of the SFMC0.1 cell are reduced compared to those of the SFM cell. This indicates that Cu doping facilitates the oxygen ion transfer steps between the YSZ electrolyte and electrode, as well as charge transfer processes at the electrode–electrolyte interface, which is consistent with the analysis of the O 1s results from XPS. Figure 5d summarizes the resistances at different frequencies. In line with the trend observed in Figure 5c, R_3_ values are significantly higher than those of R_1_ and R_2_, indicating that the processes such as CO_2_ adsorption, dissociation and diffusion at the surface are rate-limiting steps. The Rp value for SFMC0.1 is the lowest at 2.66 Ω cm^2^, while the Rp value for SFM’ is the highest at 13.57 Ω cm^2^.

**Figure 6:**
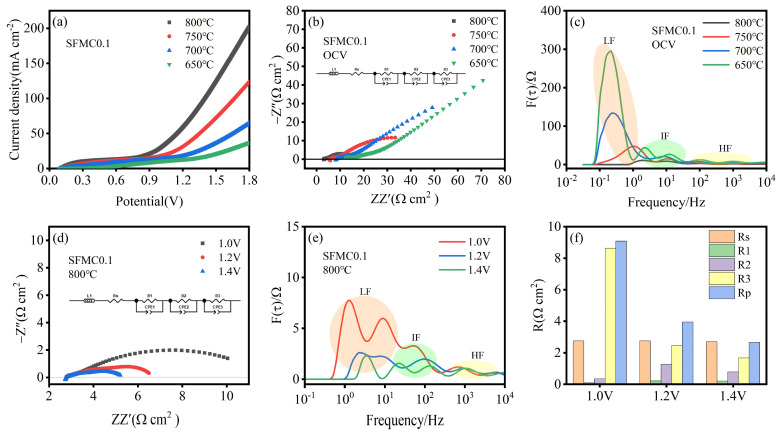
Electrochemical performance of cells with SFMC0.1 electrodes at different temperatures. (**a**) I-V curves, (**b**) EIS curves at OCV, (**c**) corresponding DRT spectra; EIS curves at different temperatures: (**d**) at different voltages, (**e**) corresponding DRT spectra, and (**f**) fitted resistance values.

Figure 6a shows the I–V curves for cells with SFMC0.1 electrodes at different temperatures. The I–V curves for all temperatures exhibit a similar trend, with current density initially increasing slowly with increasing voltage and then rising sharply. Current density increases with the operating temperature, with the highest current density of 202.20 mA cm^−2^ at 800 °C and 1.8 V, followed by 750 °C (123.53 mA cm^−2^), 700 °C (64.76 mA cm^−2^), and 650 °C (36.94 mA cm^−2^). The EIS curves at open-circuit voltage (OCV) for different temperatures are shown in Figure 6b. It can be observed that, as the temperature decreases, both the ohmic resistance and polarization resistance increase significantly. This trend is consistent with the results shown in Figure 6a. The corresponding DRT curves are presented in Figure 6c. Compared to other frequencies, the peak area in the LF region decreases most significantly as the temperature increases, and there is a tendency for it to shift towards higher frequencies. This phenomenon suggests that higher temperatures significantly enhance CO_2_ adsorption, dissociation and diffusion at the electrode surface.

Figure 6d shows the EIS curves of the cell at different voltages. The polarization resistance decreases significantly with increasing voltage, while the ohmic resistance remains almost unchanged. The corresponding DRT spectra are shown in Figure 6e. It can be observed that the resistances in the low- and medium-frequency regions decrease, indicating that applying voltage significantly influences charge transfer and the surface adsorption, diffusion and dissociation processes of the active species. According to relevant reports, cathode polarization leads to the reduction of metal ions on the electrode, generating more surface oxygen vacancies and facilitating the surface reaction process [48]. Figure 6f displays the corresponding resistances at different voltages, which are consistent with the results shown in Figure 6e. The polarization resistance at 1.4 V is 2.66 Ω·cm^2^, much smaller than at 1.0 V (9.08 Ω·cm^2^) and 1.2 V (3.95 Ω·cm^2^). The EIS-fitted parameters of SFMC0.1 electrodes at different temperatures and voltages are shown in Table S3. Table S4 compares the CO_2_ electrolysis performance between this work and other previous reports. As shown in the table, our work is better than some previous reports in terms of current density, Faradaic efficiency, and polarization resistance.

**Figure 7:**
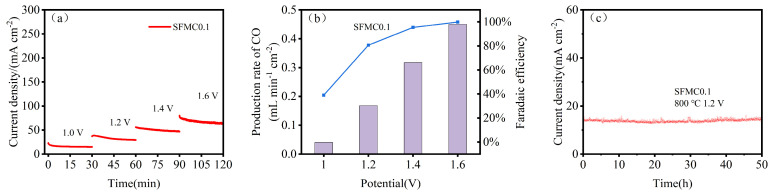
Short-term stability and performance of SFMC0.1. (**a**) Short-term stability at 1.0–1.6 V, (**b**) CO conversion rate and Faradaic efficiency and (**c**) long-term stability of the SFMC0.1 at 800 °C.

Figure 7a shows the short-term stability test results for cells based on SFMC0.1 electrodes. The current density of the cell exhibits a slight degree of degradation at high voltage. Figure 7b shows the CO yield and Faradaic efficiency of the cell at various voltages. As the applied voltage increases, the CO yield gradually increases, and the corresponding Faradaic efficiency also rises significantly. The Faradaic efficiency reaches 97.1% at 1.6 V. It should be noted that current density is influenced significantly by various factors including the experimental equipment, cell assembly and sealing status. Improving these factors can further elevate the current density level. As shown in Figure 7c, SFMC0.1 demonstrates good durability over the 50 h testing period. Some slight fluctuations in current density were observed during the test.

**Figure 8:**
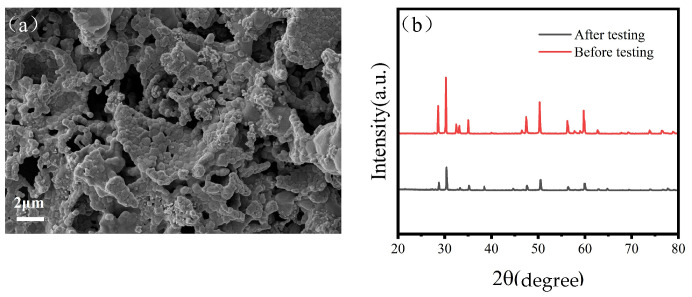
(**a**) SEM image of SFMC0.1 electrodes after testing and (**b**) XRD image of SFMC0.1 electrodes before testing and after testing.

In order to further validate the stability of the prepared SFMC0.1 electrode, Figure 8a presents the SEM image of the tested electrode. Localized particle agglomeration and coarsening are observed on its surface, indicating potential structural degradation during operation. Additionally, Figure 8b compares the XRD patterns of the SFMC0.1 electrode before and after testing. The main perovskite phase remains intact, but the weakened peak intensity may be attributed to the crystal structure at the electrode–electrolyte interface underwent changes during high-temperature testing.

### 3.4. DFT Calculations

**Figure 9:**
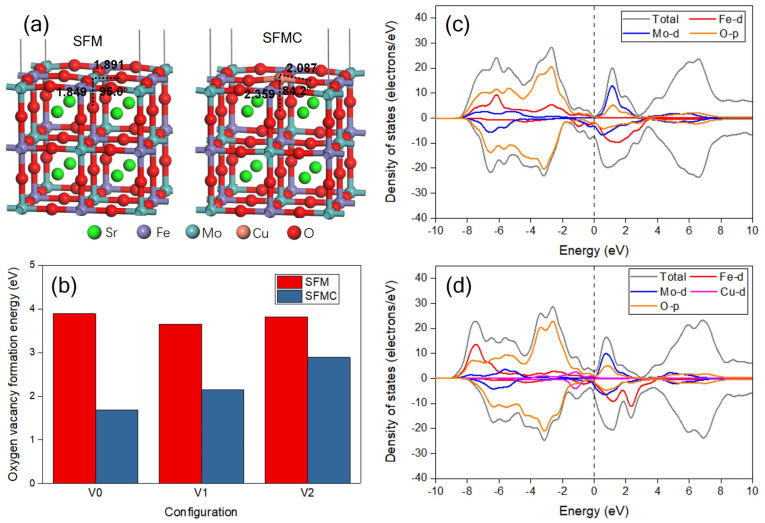
(**a**) Optimized configurations of the perfect slab models of SFM and SFMC; (**b**) oxygen vacancy formation energies of SFM and SFMC; DOS plots of (**c**) SFM and (**d**) SFMC with one surface oxygen vacancy.

Figure 9a gives the constructed perfect slabs of SFM and SFMC. As shown in the picture, the distances between the surface Mo atom and adjacent O atoms are 1.891 and 1.849 Å, respectively, and the O-Mo-O bond angle is 95.0°. As the surface Mo atom is substituted by the Cu atom, the O-Cu bond distances are extended to 2.087 and 2.359 Å, and the O-Cu-O bond angle is decreased to 84.2°. This change indicates the lattice distortion, and the enlarged distance of Cu probably facilitates the formation of oxygen vacancies. Figure S3 displays some typical defect configurations with one oxygen vacancy for SFM and SFMC after geometry optimization, and the oxygen vacancy formation energy is summarized in Figure 9b. The surface oxygen vacancy formation energy of SFM is 3.89 eV, but it decreases significantly to 1.69 eV for SFMC. The Cu doping and the formation of oxygen vacancies may enhance the intrinsic conductivity and charge carrier concentration of the catalyst, leading to moderate adsorption energy between the catalyst and reactants/intermediates, thereby synergistically improving the catalytic activity. This result confirms that the Cu substitution promotes the formation of surface oxygen vacancy, which is in accordance with the result of O 1s XPS. In addition, the lattice oxygen vacancy formation energies are also decreased, from 3.65 and 3.82 eV to 2.15 and 2.89 eV, respectively, after Cu doping, facilitating oxygen vacancy generation and enhancing ionic conductivity via O^2−^ migration pathways [46]. This phenomenon indicates that the formation of oxygen defects in a lattice are also improved, which is beneficial to the O^2−^ ion transportation across the electrode material.

Figure 9c displays the DOS plots of SFM and SFMC with one surface oxygen vacancy. In Figure 9a, the undoped SFM material shows no significant upward DOS near the Fermi level, indicating a low electron density of states in this region, which suggests that the material may have a semiconductor property. The main contributions come from the Fe-d, Mo-d and O-p orbitals. In contrast, Figure 9b shows that after Cu doping, the material exhibits a noticeable upward DOS near the Fermi level, indicating that Cu doping introduces new electron states. This can significantly enhance the intrinsic electronic conductivity of the material while increasing charge supply to the active sites of the catalytic reaction, thereby optimizing charge transfer kinetics [46]. These electronic states make a substantial contribution to the total DOS in this region. The contribution of the Cu-d orbitals near the Fermi level is evident, which may alter the electronic structure of the material, making it more metallic. In addition, after Cu doping, the increase in O-p DOS near the Fermi level may contribute to enhancing the conductivity of O^2−^ ions. These changes could have a significant impact on the material’s electrical conductivity and O^2−^ transport properties.

## 4. Conclusions

In this study, Mo ions in SFM electrode were partially substituted by Cu for CO_2_ electrolysis in SOECs, and the role of Cu substitution was revealed by experimental and theory calculation. The results show that Cu substitution induced lattice expansion and eliminated the SrMoO_4_ and Sr_2_ZrO_4_ impurity phases in the electrode and thereby enhanced the compatibility between the electrode and electrolyte. XPS results indicated that Cu doping increased the concentration of oxygen vacancies and reduced the valence states of Fe and Mo. Among various samples, the SFMC0.1 cell exhibited the largest current density and the smallest polarization resistance. At 800 °C and 1.8 V, the current density of SFMC0.1 reached 202.20 mA cm^−2^, which is a 12.8% improvement compared to SFM. At 1.4 V, the polarization resistance was 2.66 Ω·cm^2^, representing a 70.7% reduction compared to that at 1.0 V. EIS analysis demonstrated that Cu doping not only promoted CO_2_ adsorption, dissociation and diffusion processes, but improved the charge transfer and oxygen ion migration. DFT calculations confirmed that Cu doping lowered the surface and lattice oxygen vacancy formation energy of the material, thereby providing more CO_2_ active sites and facilitating oxygen ion transfer. This study offers both technical and theoretical insights for the development of new CO_2_ electrolysis electrodes in SOECs. In the future, some other factors such as cell fabrication and sealing processes should be investigated to further improve the electrode structure and cell performance. In situ/operando XRD and TEM studies will be pursued to dynamically resolve the structural evolution of the electrode during electrolysis.

## Figures and Tables

**Table 1:** Fitting results of the O 1s spectra of SFM, SFMC0.1, and SFMC0.3 powders.

Samples	Binding Energy (eV)		Relative Content (%)	
	O_lat._	O_ads._	O_lat._	O_ads._
SFM	529.3	531.0	36.9	63.1
SFMC0.1	529.8	531.0	27.9	72.1
SFMC0.3	530.8	530.9	5.6	94.4

**Table 2:** Fitting results of the Fe 2p spectra of SFM, SFMC0.1, and SFMC0.3 powders.

Samples	Binding Energy (eV)			Relative Content (%)		
	Fe^2+^	Fe^3+^	Fe^4+^	Fe^2+^	Fe^3+^	Fe^4+^
SFM	709.7	711.2	713.5	26.6	38.1	35.3
SFMC0.1	709.7	711.3	713.5	38.6	32.7	28.7
SFMC0.3	709.4	711.4	714.1	36.9	33.1	30.0

**Table 3:** Fitting results of the Mo 3d spectra of SFM, SFMC0.1, and SFMC0.3 powders.

Samples	Binding Energy (eV)		Relative Content (%)	
	Mo^5+^	Mo^6+^	Mo^5+^	Mo^6+^
SFM	231.7	232.8	2.9	97.1
SFMC0.1	231.7	235.9	16.1	83.9
SFMC0.3	231.7	229.1	6.3	93.7

## Data Availability

The original contributions presented in this study are included in the article/Supplementary Materials. Further inquiries can be directed to the corresponding authors.

## Supplementary Materials

The following supporting information can be downloaded at: https://www.mdpi.com/article/10.3390/nano15080585/s1, Figure S1: Rietveld refinement profiles of SFMC0.1 powder; Figure S2: The Williamson–Hall plot of SFM, SFMC0.1 and SFMC0.3 powder; Figure S3: Configurations of SFM and SFMC with one oxygen vacancy; Table S1: Rietveld refinement results of SFMC0.1 powder derived from XRD patterns; Table S2: The crystallite sizes of SFM, SFMC0.1 and SFMC0.3 powder were determined using the Williamson–Hall plot and the Scherrer equation; Table S3: The EIS-fitted parameters of SFMC0.1 electrodes at different temperatures and voltages; Table S4: Comparison of literature values of polarization resistance, current density and Faradaic efficiency achieved for CO_2_ electrolysis in electrolyte-supported cells under 800 °C. Refs. [15,49,50,51,52,53,54,55,56] are cited in the Supplementary Materials.
