# Supplementary material for: Effects of Cu Substituting Mo in Sr2Fe1.5Mo0.5O6−δ Symmetrical Electrodes for CO2 Electrolysis in Solid Oxide Electrolysis Cells
Source: Nanomaterials (Basel). 2025 Apr 11;15(8):585. doi: 10.3390/nano15080585 (PMC12029250; doi:10.3390/nano15080585)
Supplement: Supplementary file 1 [file nanomaterials-15-00585-s001.zip › nanomaterials-3520986-supplementary.pdf]

Supplementary Materials

Effects of Cu Substituting Mo in $Sr_2Fe_{1.5}Mo_{0.5}O_{6-\delta}$ Symmetrical Electrodes for CO_2 Electrolysis in Solid Oxide Electrolysis Cells

Wanting Tan [1], Pengzhan Hu [2], Tianxiang Feng [1], Siliang Zhao [1], Shuai Wang [1], Hui Song [2], Zhaoyu Qi [3,*] and Wenjie Li [1,4,*]

1 School of Ecology and Environment, Zhengzhou University, Zhengzhou 450001, China
2 College of Chemistry, Zhengzhou University, Zhengzhou 450001, China
3 Key Laboratory of Environmental Protection in Water Transport Engineering Ministry of Transport, Tianjin Research Institute for Water Transport Engineering, Tianjin 300456, China
4 Henan Key Laboratory of Environmental Chemistry and Low Carbon Technology, Zhengzhou 450001, China
* Correspondence: qizhy@tiwte.ac.cn (Z.Q.); wenjieli@zzu.edu.cn (W.L.)

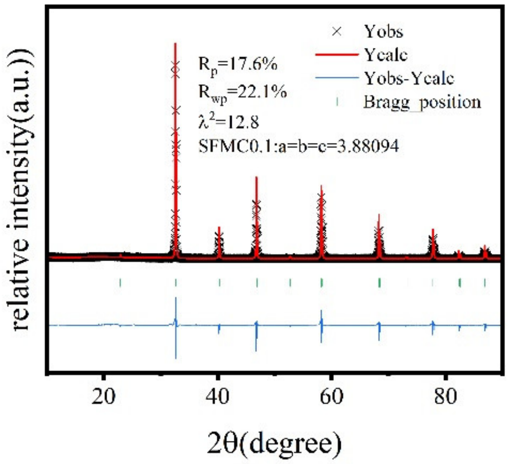

Figure S1. Rietveld refinement profiles of SFMC0.1 powder

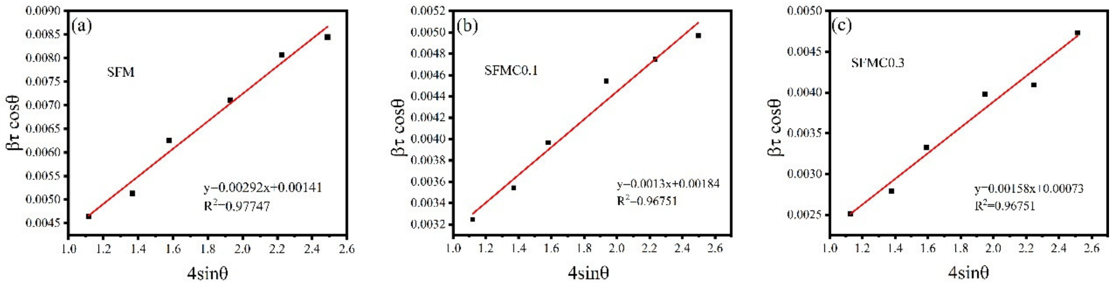

Figure S2. The Williamson-Hall plot of **(a)** SFM, **(b)** SFMC0.1 and **(c)** SFMC0.3 powder

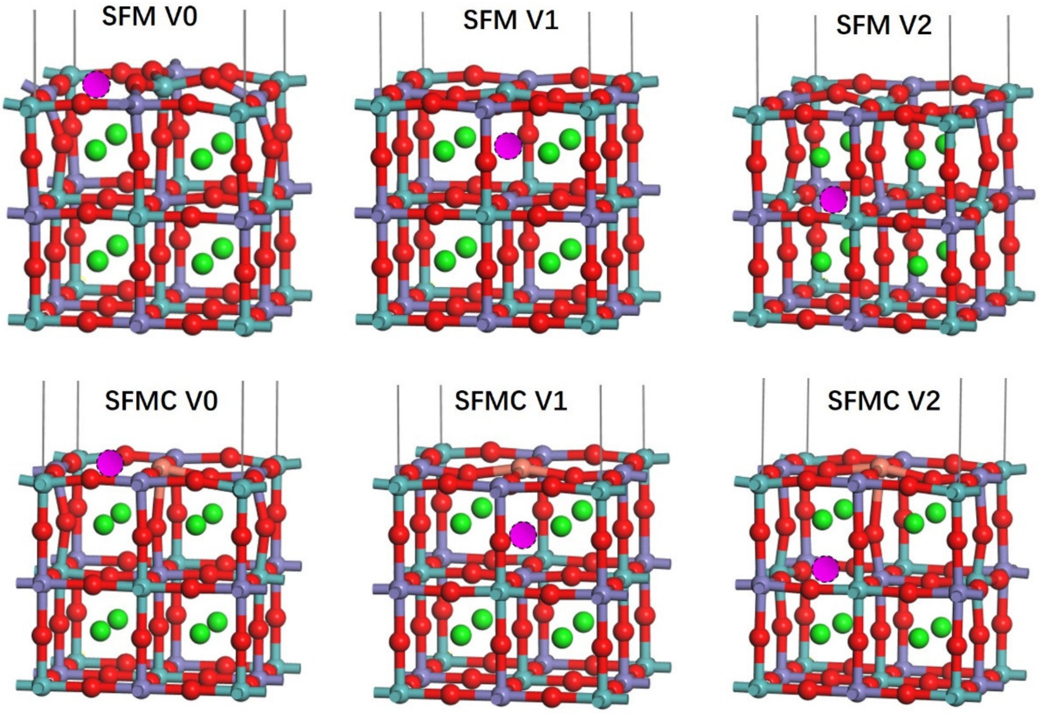

Figure S3. Configurations of SFM and SFMC with one oxygen vacancy.

Table S1 Rietveld refinement results of SFMC0.1 powder derived from XRD patterns.

Sample	R_p (%)	R_{wp} (%)	R_{exp} (%)	Space group	a (Å)	Volume (Å^3)
SFMC0.1	17.6	22.1	2.9	Pm-3m	3.88094	58.43

Table S2. The crystallite sizes of SFM, SFMC0.1 and SFMC0.3 powder were determined using the Williamson-Hall plot and the Scherrer equation

Crystallite Size(nm)	Williamson-Hall	Scherrer equation
SFM	98.3	21.5
SFMC0.1	75.4	32.5
SFMC0.3	189.7	37.8

Table S3. The EIS fitted parameters of SFMC0.1 electrodes at different temperatures and voltages

	800 °C	750 °C	700 °C	650 °C	1.0V	1.2V	1.4V
L_1	10^{-7}	10^{-7}	10^{-7}	10^{-7}	10^{-7}	10^{-7}	10^{-7}
Rs	3.04	5.29	7.22	8.84	2.75	2.75	2.71
R_1	0.47	0.15	1.63	5.58	0.10	0.21	0.23
R_2	1.95	4.58	6.95	12.65	0.35	1.27	0.76
R_3	10.16	47.45	273.04	2521.64	8.63	2.47	1.67

Table S4. Comparison of literature values of polarization resistance, current density and Faradaic efficiency achieved for CO_2 electrolysis in electrolyte-supported cells under 800 °C

Cathode	Anode	Electrolyte	polarization resistance (Ω cm^2)	Current density at 1.5 V (A cm^{-2})	Faradaic efficiency at 1.6V (%)	reference
$La_{0.75}Sr_{0.25}Cr_{0.5}Mn_{0.5}O_{3-\delta}$-SDC	$La_{0.75}Sr_{0.25}Cr_{0.5}Mn_{0.5}O_{3-\delta}$-SDC	YSZ	2.7@1.5V	0.075	58@1.5V	[1]
$La_{0.75}Sr_{0.25}Cr_{0.5}Fe_{0.5}O_{3-\delta}$-YSZ	$La_{0.8}Sr_{0.2}MnO_3$-YSZ	YSZ	4.8@1.8V	0.09	60	[2]
$La_{0.75}Sr_{0.25}Cr_{0.4}Fe_{0.5}Ti_{0.1}O_{3-\delta}$-YSZ	$La_{0.8}Sr_{0.2}MnO_3$-YSZ	YSZ	3.6@1.8V	0.075	90	[2]
$La_{0.2}Sr_{0.8}Ti_{0.9}Mn_{0.1}O_{3-\delta}$-SDC	$(La_{0.8}Sr_{0.2})_{0.95}MnO_{3-\delta}$-SDC	YSZ	3.8@1.2V	0.12	86	[3]
$La_{0.2}Sr_{0.8}TiO_{3-\delta}$-SDC	$(La_{0.8}Sr_{0.2})_{0.95}MnO_{3-\delta}$-SDC	YSZ	5.4@1.2V	0.06	60	[3]
$La_{0.2}Sr_{0.8}TiO_{3-\delta}$-SDC	$(La_{0.8}Sr_{0.2})_{0.95}MnO_{3-\delta}$-SDC	YSZ	1.73@1.6V	0.105	81.4	[4]
$Sr_{0.95}Ti_{0.9}Nb_{0.1}O_{3-\delta}$-SDC	$(La_{0.8}Sr_{0.2})_{0.95}MnO_{3-\delta}$-SDC	YSZ	2.10@1.4V	0.12	70	[5]
$Pr_{0.7}Ba_{0.3}MnO_{3-\delta}$-SDC	$Pr_{0.7}Ba_{0.3}MnO_{3-\delta}$-SDC	YSZ	1@1.6V	0.2@850 °C	87	[6]
$La_{0.7}Sr_{0.3}Cr0_{.5}Mn_{0.5}O_{3-\delta}$-SDC	$La_{0.7}Sr_{0.3}Cr0_{.5}Mn_{0.5}O_{3-\delta}$-SDC	LSGM	1.55@1.4V	0.25@850 °C	92	[7]

$Bi_{0.2}Sr_{1.8}Fe_{1.5}Mo_{0.5}O_{6-\delta}$	$Ba_{0.5}Sr0.5Co_{0.8}Fe_{0.2}O_{3-\delta}$	LSGM	0.23@1.5V	0.54	-	[8]
$La_{0.3}Sr_{1.7}Fe_{1.5}Ni_{0.1}Mo_{0.4}O_{6-\delta}$	$La_{0.3}Sr_{1.7}Fe_{1.5}Ni_{0.1}Mo_{0.4}O_{6-\delta}$	LSGM	0.13@1.5V	1.17	-	[9]
$Sr_2Fe_{1.5}Mo_{0.4}Cu_{0.1}O_{6-\delta}$-SDC	$Sr_2Fe_{1.5}Mo_{0.4}Cu_{0.1}O_{6-\delta}$-SDC	YSZ	2.66@1.4V	0.13	97.1	this work

References

1. Xu, S.; Li, S.; Yao, W.; Dong, D.; Xie, K. Direct electrolysis of CO_2 using an oxygen-ion conducting solid oxide electrolyzer based on $La_{0.75}Sr_{0.25}Cr_{0.5}Mn_{0.5}O_{3-\delta}$ electrode. *J. Power Sources* **2013**, *230*, 115-121, doi:https://doi.org/10.1016/j.jpowsour.2012.12.068.
2. Yao, W.; Duan, T.; Li, Y.; Yang, L.; Xie, K. Perovskite chromate doped with titanium for direct carbon dioxide electrolysis. *New J. Chem.* **2015**, *39*, 2956-2965, doi:https://doi.org/10.1039/C4NJ01868K.
3. Qi, W.; Gan, Y.; Yin, D.; Li, Z.; Wu, G.; Xie, K.; Wu, Y. Remarkable chemical adsorption of manganese-doped titanate for direct carbon dioxide electrolysis. *J. Mater. Chem. A* **2014**, *2*, 6904-6915, doi:https://doi.org/10.1039/C4TA00344F.
4. Ye, L.; Zhang, M.; Huang, P.; Guo, G.; Hong, M.; Li, C.; Irvine, J.T.S.; Xie, K. Enhancing CO_2 electrolysis through synergistic control of non-stoichiometry and doping to tune cathode surface structures. *Nat. Commun.* **2017**, *8*, 14785-14785, doi:https://doi.org/10.1038/ncomms14785.
5. Zhang, J.; Xie, K.; Wei, H.; Qin, Q.; Qi, W.; Yang, L.; Ruan, C.; Wu, Y. In situ formation of oxygen vacancy in perovskite $Sr_{0.95}Ti_{0.8}Nb_{0.1}M_{0.1}O_3$ (M = Mn, Cr) toward efficient carbon dioxide electrolysis. *Sci Rep* **2014**, *4*, 7082, doi:https://doi.org/10.1038/srep07082.
6. Shan, F.; Chen, T.; Ye, L.; Xie, K. Ni–doped $Pr_{0.7}Ba_{0.3}MnO_{3-\delta}$ cathodes for enhancing electrolysis of CO_2 in eolid oxide electrolytic cells. *Molecules* **2024**, *29*, 4492, doi:https://doi.org/10.3390/molecules29184492.
7. Ma, G.; Xu, Y.; Xie, K. Enhanced electrolysis of CO_2 with Metal–oxide interfaces in perovskite cathode in solid oxide electrolysis cell. *Catalysts* **2022**, *12*, 1607, doi:https://doi.org/10.3390/catal12121607.
8. Yang, M.; Yao, Z.; Liu, S.; Wang, J.; Sun, A.; Xu, H.; Yang, G.; Ran, R.; Zhou, W.; Xiao, G.; et al. Bismuth doped $Sr_2Fe_{1.5}Mo_{0.5}O_{6-\delta}$ double perovskite as a robust fuel electrode in ceramic oxide cells for direct CO_2 electrolysis. *J. Mater. Sci. Technol.* **2023**, *164*, 160-167, doi:https://doi.org/10.1016/j.jmst.2023.04.061.
9. Wang, Y.; Cui, C.; Wang, S.; Zhan, Z. Symmetrical La^{3+}-doped $Sr_2Fe_{1.5}Ni_{0.1}Mo_{0.4}O_{6-\delta}$ electrode solid oxide fuel cells for pure CO_2 electrolysis. *J. Inorg. Mater.* **2021**, *36*, 1323-1329, doi:https://doi.org/10.15541/jim20210206.
